# Dysregulated glial genes in Alzheimer's disease are essential for homeostatic plasticity: Evidence from integrative epigenetic and single cell analyses

**DOI:** 10.1111/acel.13989

**Published:** 2023-09-15

**Authors:** Yimei Cai, Tao Cui, Pengqi Yin, Paxton Paganelli, Stefano Vicini, Tingting Wang

**Affiliations:** ^1^ Department of Pharmacology & Physiology Georgetown University Medical Center Washington D.C. USA; ^2^ Interdisciplinary Program in Neuroscience Georgetown University Medical Center Washington D.C. USA; ^3^ Present address: Department of Neurology, Shanghai General Hospital Shanghai Jiao Tong University School of Medicine Shanghai China; ^4^ Present address: Department of Neurology, First Affiliated Hospital Harbin Medical University Harbin China

**Keywords:** Alzheimer's disease, *drosophila*, epigenetic, glia, histone acetylation, homeostatic plasticity, single cell transcriptome

## Abstract

Synaptic homeostatic plasticity is a foundational regulatory mechanism that maintains the stability of synaptic and neural functions within the nervous system. Impairment of homeostatic regulation has been linked to synapse destabilization during the progression of Alzheimer's disease (AD). Recent epigenetic and transcriptomic characterizations of the nervous system have revealed intricate molecular details about the aging brain and the pathogenesis of neurodegenerative diseases. Yet, how abnormal epigenetic and transcriptomic alterations in different cell types in AD affect synaptic homeostatic plasticity remains to be elucidated. Various glial cell types play critical roles in modulating synaptic functions both during the aging process and in the context of AD. Here, we investigated the impact of glial dysregulation of histone acetylation and transcriptome in AD on synaptic homeostatic plasticity, using computational analysis combined with electrophysiological methods in *Drosophila*. By integrating snRNA‐seq and H3K9ac ChIP‐seq data from the same AD patient cohort, we pinpointed cell type‐specific signature genes that were transcriptionally altered by histone acetylation. We subsequently investigated the role of these glial genes in regulating presynaptic homeostatic potentiation in *Drosophila*. Remarkably, nine glial‐specific genes, which were identified through our computational method as targets of H3K9ac and transcriptional dysregulation, were found to be crucial for the regulation of synaptic homeostatic plasticity in *Drosophila*. Our genetic evidence connects abnormal glial transcriptomic changes in AD with the impairment of homeostatic plasticity in the nervous system. In summary, our integrative computational and genetic studies highlight specific glial genes as potential key players in the homeostatic imbalance observed in AD.

## Graphical abstract

We integrated data from two studies on H3K9ac ChIP‐seq and snRNA‐seq in Alzheimer's disease (AD) patients and identified genes that are both directly targeted by H3K9ac and are cell type‐specific DEGs in AD. We studied nine glial‐specific DEGs to investigate their roles in synaptic homeostatic plasticity in Drosophila. All nine of these genes, targets of H3K9ac and glial DEGs in AD, are necessary for homeostatic plasticity, highlighting the critical role of glia in stabilizing synaptic function in AD.

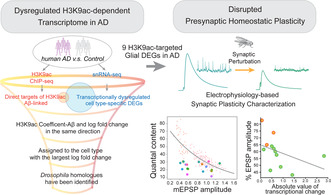

**Abbreviations**
AD: Alzheimer's Disease
Aβ42: Amyloid β42
ChIP‐seq: chromatin immunoprecipitation‐sequencing
DEG: differentially expressed gene
EPSP: excitatory postsynaptic potential
FDR: false discovery rate
GO: gene ontology
H3K9ac: acetylation at H3 lysine 9 site
mEPSP: miniature excitatory postsynaptic potential
NMJ: neuromuscular junction
OPC: oligodendrocyte progenitor cell
PHP: presynaptic homeostatic potentiation
PhTX: philanthotoxin
QC: quantal content
SAGA: Spt‐Ada‐Gcn5 acetyltransferase complex
snRNA‐seq: single nucleus RNA‐sequencing

## 1 INTRODUCTION

Alzheimer's disease (AD) is a prominent neurodegenerative disorder and the leading cause of dementia in older adults (Knopman et al., 2021). Dysfunctional synaptic activity is closely linked with AD pathology progression (Selkoe, 2002). Homeostatic plasticity plays a critical role in preserving synaptic physiology, neural activity, and overall brain function (Davis, 2006; Turrigiano, 2008). Disruptions in this mechanism can lead to neural network instability and are implicated in conditions such as epilepsy and neurodegenerative disorders (Orr et al., 2020; Styr & Slutsky, 2018; Wondolowski & Dickman, 2013). Presynaptic homeostatic potentiation (PHP) is a fundamental form of homeostatic regulation that is evolutionarily conserved from *Drosophila* to humans (Cull‐Candy et al., 1980; Davis & Muller, 2015; Plomp et al., 1992). PHP ensures stability in postsynaptic excitation by counteracting perturbations in glutamate receptors through compensatory upregulation in presynaptic neurotransmitter release (Dickman & Davis, 2009; Frank et al., 2006). Notably, PHP operates across central and peripheral nervous systems, underscoring its significance in maintaining synaptic stability (Chipman et al., 2022; Delvendahl et al., 2019; Wang et al., 2018).

Recent studies have linked shifts in the epigenetic landscape to potential causes of AD, deepening our understanding of the disease's molecular etiology (Klein et al., 2019; Lord & Cruchaga, 2014; Morabito et al., 2021; Nativio et al., 2018). Notably, single‐cell transcriptomic investigations in both AD mouse models and patient cohorts have illuminated critical roles of glia in AD pathogenesis (Grubman et al., 2019; Habib et al., 2020; Keren‐Shaul et al., 2017; Lau et al., 2020; Zhou et al., 2020). While neuronal deficits in AD have been the subject of extensive research, there is growing evidence underscoring the important function glial cells have in modulating synaptic transmission and plasticity, both under normal conditions and in the diseased brain (Chung et al., 2015; Hong et al., 2016; Keren‐Shaul et al., 2017; Stogsdill & Eroglu, 2017). Our previous work has shown that glial epigenetic signaling, as well as interactions between glia and neurons, are fundamental for inducing and sustaining synaptic homeostatic plasticity in *Drosophila* (Wang et al., 2014; Wang et al., 2020). Yet, the influence of abnormal epigenetic and transcriptomic shifts in glial cells on synaptic homeostatic plasticity within the context of AD remains largely unexplored.

In this study, we investigated how the aberrant histone acetylation and glial transcriptomic shifts associated with AD affect synaptic homeostasis. By employing computational methods, we integrated data from previously published ChIP‐seq and snRNA‐seq datasets (Klein et al., 2019; Mathys et al., 2019). Through this, we identified cell type‐specific differentially expressed genes (DEGs) in post‐mortem AD brains, which were consistently regulated by histone acetylation. Following this, we examined the function of these glial‐specific DEGs on homeostatic plasticity through both genetic and electrophysiological methods in *Drosophila* models. Notably, all nine glial‐specific DEGs we analyzed were indispensable for PHP. This emphasizes the pivotal role glia play in homeostatic regulation and the preservation of synaptic stability.

## 2 CELL TYPE‐SPECIFIC DYSREGULATION OF H3K9AC‐TARGET GENES IN AD

To understand the molecular interplay between aberrant histone acetylation, transcriptomic changes in AD, and homeostatic plasticity, we began by pinpointing genes affected transcriptionally by abnormal histone acetylation in AD. We were particularly drawn to the dysregulation at the histone H3 lysine nine site (H3K9ac) for several reasons. First, prior studies underscored an anomalous shift in H3K9ac‐dependent epigenetic modulation within AD brains, but the direct consequences of this dysregulation on synaptic functions are yet to be elucidated (Klein et al., 2019; Santana et al., 2022; Tasaki et al., 2019). Second, our previous research showed that the Spt‐Ada‐Gcn5 acetyltransferase (SAGA) complex‐driven histone acetylation at H3K9ac in glia is crucial for the glial‐neuron interactions necessary for synaptic homeostatic plasticity (Wang et al., 2020). Third, in a subsequent study exploring glial epigenetic regulation in *Drosophila* AD models, we observed a pronounced reduction in H3K9ac levels within glial nuclei in transgenic models of Amyloid β42 (Aβ42). Additionally, overexpression of human Aβ42 in neurons (but not tau) led to PHP deficits (Yin et al., 2023). This indicates that glial cells may detect changes in Aβ levels, prompting abnormal H3K9ac‐mediated signaling that impairs PHP. Given these insights, our study focused on investigating H3K9ac‐driven transcriptomic changes across various cell types in AD, with a spotlight on glia.

**FIGURE 1:**
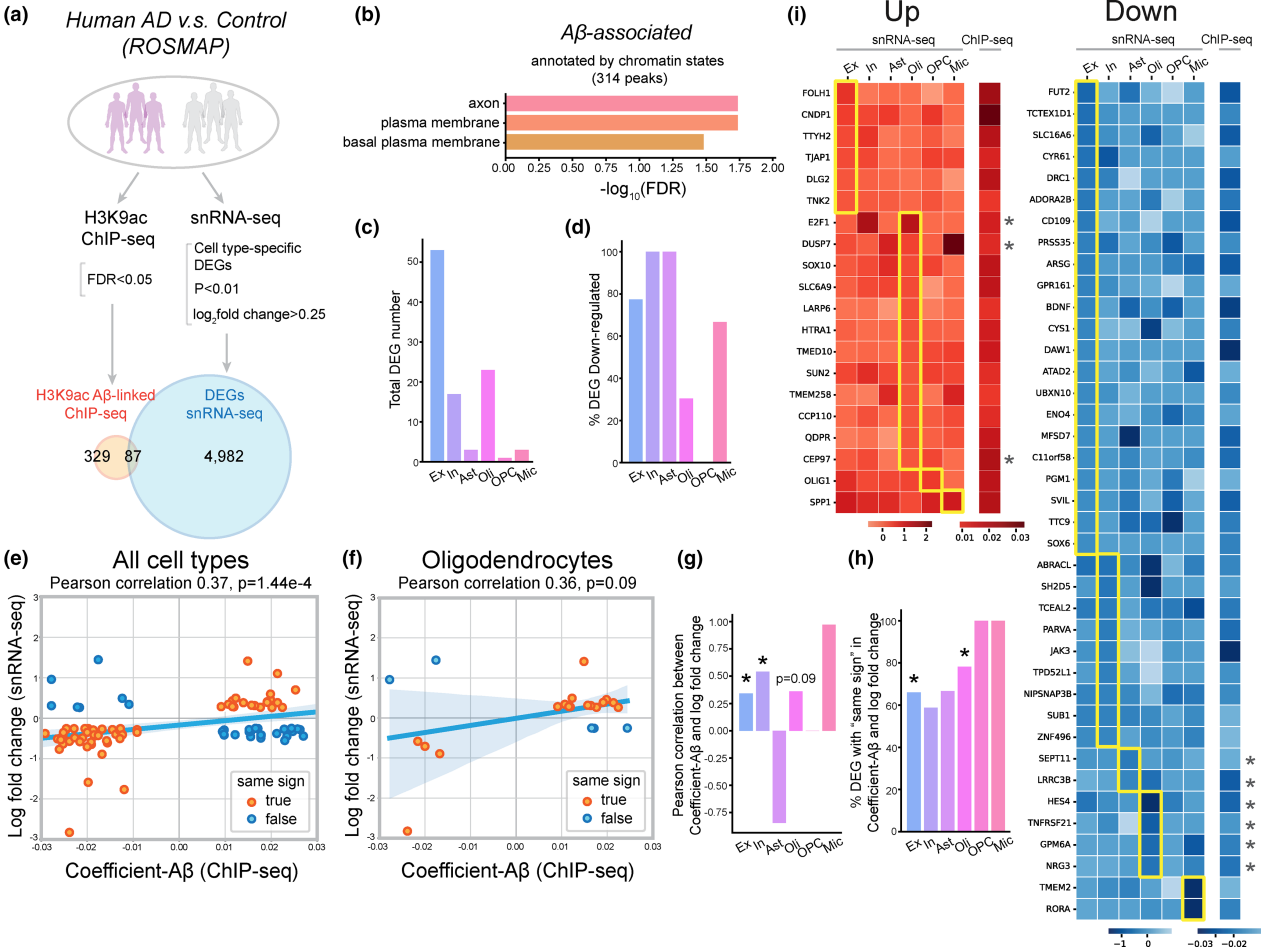
H3K9ac‐target Genes Are Differentially Regulated in Various Cell Types in AD. (a) Schematic to show an integrative analysis comparing H3K9ac‐target genes (ChIP‐seq) and single cell transcriptomic changes (snRNA‐seq) in human AD patients versus controls. Criteria for gene selection and the total number of overlapping unique genes are shown. (b) GO terms identified from genes (annotated by chromatin states as in the published study) that are associated with Aβ in the H3K9ac ChIP‐seq dataset. (c, d) The total number (c) and percentage of downregulated genes (d) that are both H3K9ac targets and cell type‐specific DEGs in excitatory neuron (Ex), inhibitory neuron (In), astrocyte (Ast), oligodendrocyte (Oli), oligodendrocyte progenitor cell (OPC), and microglia (Mic). (e, f) Correlations between coefficient Aβ and log_2_fold change of DEGs in all cell types (e) and oligodendrocytes (f). DEGs that have coefficient Aβ and log_2_fold change with the “same sign” are shown in red. (g, h) The correlation between coefficient Aβ and log_2_fold change of DEGs (g) and percentage of DEGs that have coefficient Aβ and log_2_fold change with the “same sign” (h) in different cell types. *p < 0.05; Pearson correlation for (g) and two‐sided binomial test for (h). (i) Transcriptional log_2_fold change (snRNA‐seq) and coefficient Aβ (ChIP‐seq) for genes that are dysregulated (up and downregulated) and have the “same sign.” Cell type‐specific DEGs are highlighted with yellow boxes. When a gene is a DEG in multiple cell types, it is assigned to the cell type with the largest absolute value of log_2_fold change. Glial DEGs that have homologues in *Drosophila* and are selected for PHP characterization are labeled with asterisks.

We performed an integrative analysis by combining a previously published H3K9ac ChIP‐seq dataset (Klein et al., 2019) with a snRNA‐seq dataset (Mathys et al., 2019). Both datasets originated from the prefrontal cortex of post‐mortem brains and were part of the same cohort studies on aging and dementia, specifically the ROSMAP project (Figure 1a). Recent studies underline that the accumulation of Aβ not only leads to glial activation but also markedly compromises glial functionality (Huffels et al., 2023; Sierksma et al., 2020; Zeng et al., 2023). In our previous work, we observed that Aβ42 overexpression caused reduction in glial H3K9ac and led to PHP impairment in *Drosophila* AD models (Yin et al., 2023). Therefore, we centered our analysis on the H3K9ac peaks associated with Aβ. First, we analyzed these H3K9ac peaks in the ChIP‐seq dataset, using the criteria delineated in the original publication (FDR < 0.05). A subsequent Gene Ontology (GO) assessment of the 329 genes, which were annotated based on chromatin states from the original study, pinpointed pathways that were specifically related to Aβ (Figure 1b; Figure S1a). It is worth noting that the genes associated with Aβ play crucial roles in modulating signaling processes related to axon and plasma membrane activities (Figure 1b; Figure S1a).

In our subsequent analysis, we aimed to identify DEGs targeted by H3K9ac that exhibit dysregulation across various cell types in the AD brain. Drawing from the published snRNA‐seq study, we found a total of 4982 AD‐associated DEGs (Figure 1a). By overlapping these DEGs with the 329 Aβ‐associated H3K9ac‐target genes sourced from the ChIP‐seq dataset, we sought to determine cell type‐specific transcriptomic alterations. We found that 100 H3K9ac‐targeted cell type‐specific DEGs (representing 87 distinct genes) displayed dysregulation, either being up‐ or downregulated in AD (Figure 1a,c). Notably, the most prominent dysregulations were evident in excitatory neurons, inhibitory neurons, and oligodendrocytes (Figure 1c). A detailed breakdown revealed that the majority of DEGs in excitatory neurons (77%), inhibitory neurons (100%), and microglia (67%) were downregulated. In contrast, 70% of DEGs specific to oligodendrocytes showed upregulation (Figure 1d). The opposing transcriptional changes observed in neurons and oligodendrocytes suggest that the downregulation in gene expression within neurons could be a consequence of disease pathology, while the upregulation in gene expression in oligodendrocytes could potentially indicate a compensatory regulatory response linked to AD. These findings underscore the cell type‐specific changes of H3K9ac‐targeted DEGs in the context of AD.

## 3 TRANSCRIPTOMIC CHANGES CORRELATE WITH AΒ‐LINKED H3K9AC ALTERATIONS IN AD

To assess the impact of Aβ pathology‐related H3K9ac modifications on cell‐specific gene transcription in AD, we probed the correlation between coefficient Aβ (from ChIP‐seq) and cell type‐specific transcriptomic shifts (from snRNA‐seq). Coefficient Aβ denotes the extent to which H3K9ac peak reads in the ChIP‐seq dataset are influenced by the severity of Aβ pathology. Specifically, a positive coefficient Aβ indicates that H3K9ac peak reads vary in the same direction with the overall amyloid load, and an elevated absolute value suggests that a given amyloid burden induces a more pronounced change in H3K9ac peak reads (Klein et al., 2019). We conducted a correlation analysis between coefficient Aβ and the transcriptional log fold change for each of the identified H3K9ac‐targeted DEGs (a total of 100 DEGs). A significant positive correlation was found between coefficient Aβ and transcriptional fold change across all cell types (Pearson correlation = 0.37, *p* = 1.44e‐4, Figure 1e). When we examined this correlation within specific cell types, we found significant correlations in both excitatory and inhibitory neurons (Figure 1g). Although statistical significance was not achieved for astrocytes, oligodendrocyte progenitor cells (OPCs), and microglia, possibly due to the limited number of DEGs, a Pearson correlation of 0.36 was observed for oligodendrocytes (*p* = 0.09; Figure 1f,g).

As H3K9ac typically associates with chromatin openness and active transcription (Karmodiya et al., 2012), the observed positive Pearson correlation between coefficient Aβ and transcriptional fold change across different cell types suggests that these two metrics might both be coherently regulated in response to the severity of Aβ accumulation. To further probe this, we examined the number of genes showing transcriptional log fold change that align directionally with coefficient Aβ, termed as the “same sign” (Figure 1e,f,h). Elaborating on the intricate relationship we outlined: when both coefficient Aβ and log fold change are negative, Aβ ↑ (increase) → H3K9ac ↓ (decrease) → chromatin openness ↓ (decrease) → gene expression ↓ (decrease). Conversely, when both coefficient Aβ and log fold change are positive, Aβ ↑ (increase) → H3K9ac ↑ (increase) → chromatin openness ↑ (increase) → gene expression ↑ (increase). From this illustration, it is evident that, depending upon the respective “signs” of the coefficient Aβ and log fold change, an incremental increase in Aβ levels can result in either the up‐ or downregulation of gene expression.

Remarkably, across all examined cell types, 69 out of the 100 DEGs (or 69%) displayed “same sign” in their transcriptional log fold change and coefficient Aβ values (Figure 1e,h,i; Figure S1b, with detailed data in Tables S1 and S2). Notably, previously identified AD risk factors, including SPP1, have been discovered as genes with “same sign.” To determine if this correlation was statistically significant, we conducted a two‐sided binomial test, comparing the observed percentage of genes with the “same sign” against 50% (the likelihood if signs were assigned randomly). This analysis revealed that 66% of DEGs in excitatory neurons (*p* = 0.03) and a striking 78% in oligodendrocytes (*p* = 0.01) displayed this “same sign” trait (Figure 1f,g). Given that coefficient Aβ essentially represents the impact of Aβ pathology on H3K9ac, this significant overrepresentation of genes with the “same sign” underscores the positive co‐regulation between H3K9ac dysregulation and the transcriptional abnormalities in AD.

## 4 GLIAL DEGS IDENTIFIED IN AD ARE ESSENTIAL FOR PHP IN *DROSOPHILA*

**FIGURE 2:**
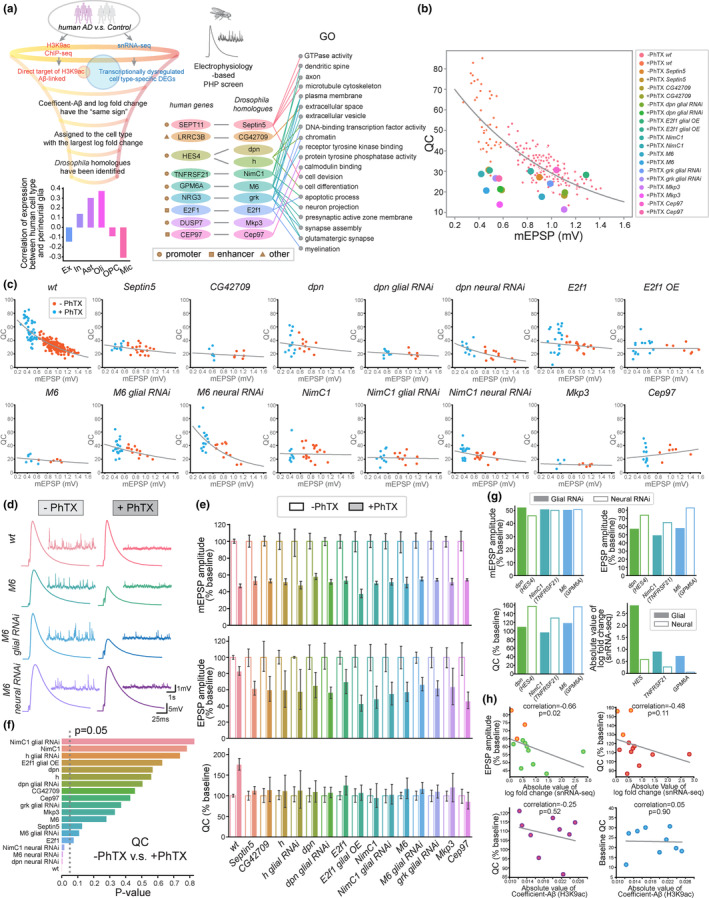
Glial DEGs that are direct targets of H3K9ac control PHP. (a) Schematic to show criteria used to select genes for the electrophysiology‐based genetic screen in *Drosophila* (upper left panel). Genes that are H3K9ac targets, cell type‐specific DEGs, acquire coefficient Aβ and transcriptional log_2_fold change with the “same sign,” and have identified *Drosophila* homologues are selected for the electrophysiological characterization. If a gene is a DEG in multiple cell types, it is assigned to the cell type with the largest absolute value of log_2_fold change. The Spearman correlation between the expression of the top 20 highest expressed human “synaptic” genes in various cell types and the expression of their homologues in *Drosophila* perineurial glia (lower left panel). Human gene names, corresponding homologues in *Drosophila*, and GO terms associated with human glial DEGs that are selected for electrophysiological screen are shown. H3K9ac peaks are in the promoter (circle), enhancer (square), or other (triangle) regions of the human genes (right panel). (b) The relationship between quantal content (QC) and mEPSP amplitude in the presence (+PhTX) and the absence of philanthotoxin (−PhTX) is shown for the *wild‐type* (*wt*), *Septin5* mutant (*Septin5*), *CG42709* mutant (*CG42709*), *NP6293‐Gal4>UAS‐dpn RNAi* (*dpn glial RNAi*), *Repo‐Gal4>UAS‐E2f1* (*E2f1 glial OE*), *NimC1* mutant (*NimC1*), *M6* mutant (*M6*), *NP6293‐Gal4>UAS‐grk RNAi* (*grk glial RNAi*), *Mkp3* mutant (*Mkp3*), and *Cep97* mutant (*Cep97*). For the *wild‐type*, each data point (small dots) represents one cell. The average QC and mEPSP amplitude (large dots) are shown for each mutant genotype. (c) QC and mEPSP amplitude in the presence (+PhTX) and the absence of philanthotoxin (−PhTX) are shown for the *wild‐type* (*wt*), *Septin5* mutant (*Septin5*), *CG42709* mutant (*CG42709*), *dpn* mutant (*dpn*), *NP6293‐Gal4>UAS‐dpn RNAi* (*dpn glial RNAi*), *Ok371‐Gal4>UAS‐dpn RNAi* (*dpn neural RNAi*), *E2f1* mutant (*E2f1*), *Repo‐Gal4>UAS‐E2f1* (*E2f1 glial OE*), *M6* mutant (*M6*), *NP6293‐Gal4>UAS‐M6 RNAi (M6 glial RNAi)*, *Ok371‐Gal4>UAS M6 RNAi* (*dpn neural RNAi*), *NimC1* mutant (*NimC1*), *NP6293‐Gal4>UAS‐NimC1 RNAi* (*NimC1 glial RNAi*), *Ok371‐Gal4>UAS‐NimC1 RNAi* (*NimC1 neural RNAi*), *Mkp3* mutant (*Mkp3*), and *Cep97* mutant (*Cep97*). Data points in the plot represent individual cells. Exponential fit lines for the datapoints are shown in gray. (d) Representative EPSP and mEPSP traces in the *wild‐type* (*wt*), *M6* mutant (*M6*), *NP6293‐Gal4>UAS‐M6 RNAi* (*M6 glial RNAi*), *Ok371‐Gal4>UAS‐M6 RNAi* (*M6 neural RNAi*), in the absence (−PhTX) and the presence of philanthotoxin (+PhTX). (e) mEPSP amplitude, EPSP amplitude, and presynaptic release (QC) in the presence (+PhTX, filled bars) and absence of philanthotoxin (−PhTX, open bars) are shown. The data for each genotype are presented as the percentage in PhTX compared to the same genotype recorded in the absence of PhTX. Genotypes and sample sizes: *wild‐type* (*wt*, *n* = 167, 43 for −PhTX and +PhTX respectively), *Septin5* mutant (*Septin5*, *n* = 19, 11), *CG42709* mutant (*CG42709*, *n* = 7, 6), *h* mutant (*h*, *n* = 8, 7), *NP6293‐Gal4>UAS‐h RNAi* (*h glial RNAi*, *n* = 4, 9), *dpn* mutant (*dpn*, *n* = 10, 12), *NP6293‐Gal4>UAS‐dpn RNAi* (*dpn glial RNAi*, *n* = 7, 9), *E2f1* mutant (*E2f1*, *n* = 17, 20), *Repo‐Gal4>UAS‐E2f1* (*E2f1 glial OE*, *n* = 7, 15), *NimC1* mutant (*NimC1*, *n* = 18, 9), *NP6293‐Gal4>UAS‐NimC1 RNAi* (*NimC1 glial RNAi*, *n* = 11, 10), *M6* mutant (*M6*, *n* = 6, 6), *NP6293‐Gal4>UAS‐M6 RNAi* (*M6 glial RNAi*, *n* = 13, 21), *NP6293‐Gal4>UAS‐grk RNAi* (*grk glial RNAi*, *n* = 10, 14), *Mkp3* mutant (*Mkp3*, *n* = 6, 5), and *Cep97* mutant (*Cep97*, *n* = 7, 13). Data are shown as mean ± SEM. (f) *p*‐values for QC of each genotype in the presence of PhTX compared to the same genotype recorded in the absence of PhTX. Student's *t*‐test; dashed line shows *p* = 0.05. (g) The percentage mEPSP amplitude, EPSP amplitude, and presynaptic release (QC) in the presence of PhTX compared to the values in absence of PhTX for the perineurial glial (*NP6293‐Gal4*, filled bars) or neural‐specific (*Ok371‐Gal4*, empty bars) knockdown of *dpn*, *NimC1*, and *M6* by RNAi. Genotypes and sample sizes: *NP6293‐Gal4>UAS‐dpn RNAi* (*dpn glial RNAi*, *n* = 7, 9 for −PhTX and +PhTX respectively), *Ok371‐Gal4>UAS‐dpn RNAi* (*dpn neural RNAi*, *n* = 11, 11), *NP6293‐Gal4>UAS‐NimC1 RNAi* (*NimC1 glial RNAi*, *n* = 11, 10), *Ok371‐Gal4>UAS‐NimC1‐RNAi* (*NimC1 neural RNAi*, *n* = 15, 16), *NP6293‐Gal4>UAS‐M6‐RNAi* (*M6 glial RNAi*, *n* = 13, 21), and *Ok371‐Gal4>UAS‐M6 RNAi* (*M6 neural RNAi*, *n* = 11, 15). The absolute values of transcriptional log_2_fold change of human *HES4*, *TNFRSF21*, *GPM6A* in pathological condition compared to non‐pathological condition. Note the largest absolute log_2_fold change in glial (filled bars) versus neuronal (open bars) cell types are shown. (h) Spearman correlations for the percentage EPSP amplitude and percentage QC of each genotype (in the presence of PhTX compared in the absence of PhTX) to the absolute values of transcriptional log_2_fold change and H3K9ac coefficient‐Aβ. Data points for neuronal‐specific knock down of *dpn*, *NimC1*, and *M6* are shown in orange in upper panels. The % EPSP amplitude versus snRNA‐seq absolute log_2_fold change (upper left panel), % QC versus snRNA‐seq absolute log_2_fold change (upper right panel), % QC versus absolute coefficient Aβ (lower left panel), and the baseline QC versus absolute coefficient Aβ (lower right) are shown.

To explore the potential association between impaired homeostatic plasticity and the concurrent anomalies in H3K9ac and transcriptomes within AD, we set forth a series of selection criteria for a genetic screen in *Drosophila* (Figure 2a). We utilized the following sequential criteria:

1. The gene is a direct target of H3K9ac associated with Aβ.
2. The gene is a dysregulated cell type‐specific DEG in AD.
3. Both the coefficient Aβ and the gene's transcriptional log fold change display the “same sign” in AD, suggesting H3K9ac and transcriptomic changes are coherently modulated.
4. If a gene is a DEG across multiple cell types, it is assigned to the cell type where it has the highest magnitude of transcriptional log fold change (absolute value).
5. There is an identified *Drosophila* homologue for the gene, signifying evolutionary conservation from *Drosophila* to humans.

Applying the first 4 criteria, we pinpointed 69 DEGs that fulfill these conditions (Figures 1e,i and 2a; Tables S1 and S2). Moreover, our data analysis indicated the majority of DEGs to be downregulated in AD (Figure 1d). Building on this, we selected all the downregulated glial‐specific DEGs (8 DEGs) that had direct *Drosophila* homologues (six meet all the criteria) and added three upregulated glial DEGs to our list for an in‐depth electrophysiological examination (Figures 1i and 2a).

The *Drosophila* neuromuscular junction (NMJ), a glutamatergic synapse, offers a powerful platform for investigating synaptic transmission and PHP through genetic and electrophysiological methods (Davis & Muller, 2015; Dickman & Davis, 2009; Muller et al., 2011). At the NMJ, three distinct peripheral glial cells—wrapping glia, subperineurial glia, and perineurial glia—interact with both the presynaptic motoneurons and postsynaptic muscles (Wang et al., 2020; Yildirim et al., 2019). This unique setup aids in decoding the intricacies of glia–neuron communications essential for PHP. Our earlier work unveiled the crucial role of perineurial glia's secretory function in PHP. There is a transcriptional upregulation of the extracellular matrix molecule, Multiplexin, in these glial cells during PHP, which is triggered by chronic inhibition of postsynaptic glutamate receptor activity, and is mediated by the SAGA complex‐dependent H3K9ac (Wang et al., 2020). These results emphasize glial signaling molecules' central role in modulating neuronal activities during PHP. Consequently, we sought to systematically examine the regulatory roles of glial‐specific DEGs (from computational analysis) in PHP, through genetic and electrophysiological examinations at the fly NMJ.

While the DEGs were identified from distinct glial cell types (astrocytes, microglia, oligodendrocytes, and OPCs) in the human brain, we first sought to determine if the perineurial glia in *Drosophila* exhibit activities similar to those of mammalian glial cells in terms of regulating synaptic functions. To this end, we conducted a computational analysis to assess whether genes linked with synaptic functions in various human glial cell types are also “enriched” and show correlative expression in *Drosophila* perineurial glia. We analyzed a scRNA‐seq dataset from *Drosophila* brains (Davie et al., 2018) and a snRNA‐seq dataset from human brains (non‐pathological, [Mathys et al., 2019]) and identified genes that possess “synaptic” functions as defined by GO terms with the keyword “synaptic.” We discovered that for the top 20 (we observed consistent results, as detailed below, when analyzing either the top 30 or 40 genes) most highly expressed synaptic‐related human genes in each cell type (excitatory neurons, inhibitory neurons, astrocytes, microglia, oligodendrocytes, and OPCs), all the corresponding *Drosophila* homologues are expressed in perineurial glia.

Next, we conducted a gene Expression Weighted Cell‐type Enrichment (“enrichment,” Skene & Grant, 2016) analysis to determine whether the *Drosophila* homologues of the top 20 “synaptic” genes expressed in human glial cell types are represented in perineurial glia. The “enrichment” refers to the likelihood of the top 20 *Drosophila* homologues within a specific human cell type displaying higher expression levels in perineurial glia than what would be expected by chance. We found that, in perineurial glia, the *Drosophila* homologues of top 20 human “synaptic” genes exhibited higher expressions than 99.8% of randomly sampled expressed genes in astrocytes, 95.7% in microglia, and 87.7% in oligodendrocytes. These findings suggest that perineurial glia play roles in regulating synaptic properties, which further underscores the potential synaptic functional parallels between these cell types.

Furthermore, for the top 20 most highly expressed human “synaptic” genes, we found that correlations between human expression levels (in various cell types) and *Drosophila* homologue expression levels in perineurial glia are positive for oligodendrocytes (Spearman correlation = 0.37, *p* = 0.11) and astrocytes (0.30, *p* = 0.20), while negative for excitatory neurons (−0.14, *p* = 0.54) and mild for inhibitory neurons (0.13, *p* = 0.13). Even though the p‐values did not reach the conventional 0.05 significance level, these findings nonetheless suggest a directional relationship between the expression levels of “synaptic” genes in *Drosophila* perineurial glia and those in human astrocytes and oligodendrocytes (Figure 2a, bottom panel). Additionally, there exists a positive correlation between the expression levels of “synaptic” genes in oligodendrocytes (Spearman correlation = 0.40, *p* = 0.08) and astrocytes (0.27, *p* = 0.25), and the percentage of perineurial glia expressing these genes (Figure S2a). Therefore, even though *Drosophila* perineurial glia do not share identical cell identities with mammalian glia in the central nervous system, it is plausible that the mechanisms for regulating synaptic functions are conserved.

Next, we examined the GO terms and found that the glial DEGs included in the genetic screen are generally involved in regulating the functions associated with axon, dendritic spine, synapse, myelination, chromatin, extracellular space, and apoptotic process (Figure 2a). Notably, these GO terms align with those associated with Aβ in the H3K9ac ChIP‐seq dataset (Figure 1b). To further understand the functions of these DEGs in regulating PHP, we investigated the rapid induction of PHP (Frank et al., 2006). We assessed PHP in loss‐of‐function genetic mutants of *Septin5*, *CG42709*, *h*, *dpn*, *E2f1*, *NimC1*, *M6*, *Mkp3*, and *Cep97*. In order to examine whether the identified DEGs function in glia for PHP, we knocked down *h*, *dpn*, *NimC1*, *M6*, and *grk* specifically in perineurial glia using RNAi strategies. Taking into account the expression of human *E2F1* is upregulated in oligodendrocytes in AD, we also explored the impact of overexpressing *E2f1* in glial cells on PHP in *Drosophila*. Moreover, as an important control, we assessed whether motoneuron‐specific knockdown of *dpn*, *NimC1*, and *M6* affect PHP (Figure 2b–e).

Bath application of philanthotoxin (PhTX) significantly reduced the mEPSP amplitude in all genotypes (Figure 2b–e). We calculated quantal content (QC) as the ratio of EPSP amplitude to mEPSP amplitude, using it as an estimate for the number of vesicles released from presynaptic terminals. In the *wild‐type*, a decrease in mEPSP amplitude is associated with an increase in presynaptic release, as inferred from QC values. This relationship indicates a negative correlation between mEPSP amplitude and QC, underscoring a compensatory mechanism that facilitates neurotransmitter release in the presence of PhTX (Figure 2b,c). Quantitatively, a significant increase in presynaptic release, as measured by QC, counterbalances the decrease in mEPSP amplitude, ensuring that postsynaptic excitation remains at the baseline value observed in the *wild‐type* (see Figure 2d,e). Conversely, there is no change in QC and the average EPSP amplitude is significantly decreased in all mutant alleles of *Septin5*, *CG42709*, *h*, *dpn*, *E2f1*, *NimC1*, *M6*, *Mkp3*, and *Cep97*, as well as perineurial glial‐specific knockdown of *h*, *dpn*, *NimC1*, *M6*, and *grk* (Figure 2b–e). Interestingly, both loss‐of‐function and gain‐of‐function (glial overexpression) of *E2f1* block PHP, highlighting a dosage‐sensitive activity of *E2f1* in synaptic homeostasis (Figure 2b–e). Notably, the negative correlation between QC and mEPSP amplitude is absent in all genetic, perineurial glial‐specific RNAi knockdown, and overexpression mutants that were tested, suggesting that PhTX‐induced homeostatic compensation is impaired when the expression of these glial DEGs is perturbed (Figure 2b,c). Taken together, we demonstrated that H3K9ac‐target genes that are dysregulated in glia in AD are critical for homeostatic plasticity in *Drosophila*.

While human *HES4* (*Drosophila dpn*) and *TNFRSF21* (*Drosophila NimC1*) showed the most significant transcriptional changes in oligodendrocytes (Figure 1i), they were identified as neuronal DEGs in AD as well. Thus, we decided to further explore whether they function in neurons or glia for PHP. As a control, we also examined the neuronal role of *M6* (human *GPM6A*, a DEG exclusively in oligodendrocytes) in PHP. We discovered that when we knockdown *dpn* and *NimC1* in motoneurons, PHP is still expressed, albeit to a lesser degree than in the *wild‐type* control (Figure 2c–g). Interestingly, PHP is completely normal when *M6* is knocked down specifically in motoneurons (Figure 2c–g). There is a significant increase in neurotransmitter release when these three genes were knockdown specifically in motoneurons (Figure 2f). Moreover, we analyzed the percentage EPSP amplitude and QC in the presence of PhTX compared to the basal conditions, when *dpn*, *NimC1*, and *M6* were knocked down specifically in perineurial glia or motoneurons (Figure 2g). We found that there was a more pronounced reduction in EPSP amplitude and a smaller increase in QC in the presence of PhTX, when these genes were specifically knocked in perineurial glia than in neurons (Figure 2g). These findings suggest that *dpn*, *NimC1*, and *M6* primarily operate in glia for synaptic homeostatic plasticity. The impairment of PHP is consistent with the observation of greater transcriptional dysregulation of *dpn*, *NimC1*, and *M6* in human glial cell types than in neurons (Figure 2g).

Finally, we examined whether the % EPSP amplitude and QC in PHP (normalized to baseline values in the absence of PhTX) is quantitatively correlated with the magnitude of transcriptional fold change and coefficient Aβ in AD. We found that the % EPSP amplitude and QC in mutants in the presence of PhTX exhibited negative correlations with the magnitude of transcriptional log fold change in AD (absolute value, Spearman correlation = −0.66, *p* = 0.02 for EPSP amplitude, −0.37, *p* = 0.11 for QC, Figure 2h). The % QC in the presence of PhTX also showed a negative correlation with the absolute value of coefficient Aβ (Spearman correlation = −0.25), while QC at baseline in the absence of PhTX was not correlated with coefficient Aβ (Spearman correlation = 0.05, Figure 2h). Given that limited number of genes (nine genes) were used in the analysis, only the correlation between the % EPSP amplitude and the absolute value of transcriptional log fold change is statistically significant (*p* = 0.02). Nonetheless, these findings underscore that the extent of PHP impairment (and not baseline synaptic transmission deficits) correlates with the extent of H3K9ac dysregulation and transcriptomic shifts in AD.

In conclusion, by integrating H3K9ac ChIP‐seq with snRNA‐seq datasets from AD patients, we identified genes targeted by H3K9ac that exhibit transcriptional dysregulation across various cell types in AD. Leveraging *Drosophila* models, we investigated nine glial‐specific DEGs and found that each of them is necessary to PHP. Tissue‐specific RNAi knockdown studies suggest these genes primarily function in perineurial glia to regulate PHP. Notably, while conventional gene discovery approaches typically yield a hit rate of 3%–4% in screens partially biased towards studying synaptic molecule functions (Dickman & Davis, 2009; Muller et al., 2011), and 1.4% in unbiased screens (Hauswirth et al., 2018) to identify genes essential for PHP, our study found that 100% of the genes we tested are required in regulating PHP. This underscores the robustness of computational analysis‐driven gene discovery in understanding the molecular mechanisms of synaptic function.

Our results suggest an important role of PHP in maintaining synaptic stability in AD, and we have identified nine genes connecting AD‐related epigenetic and transcriptomic shifts to synaptic instability. The genes we have identified serve as a valuable resource for further investigations into the mechanisms of synaptic destabilization in neurodegenerative diseases. A notable finding was the inverse correlation between the % EPSP amplitude when PhTX is present (normalized to the baseline value) and the magnitude of transcriptional log fold changes in AD (Figure 2h). As the homeostatic compensation deteriorates, the EPSP amplitude diminishes in the presence of PhTX. This indicates that more pronounced transcriptional changes in AD are linked to greater disruptions in PHP. This discovery presents, for the first time, a quantitative association between the level of PHP impairment and the scale of transcriptional abnormalities. Notably, while this correlation does not directly clarify the cell type‐specific roles of these genes in PHP, it emphasizes the broader relationship between PHP dysfunction and transcriptional dysregulation. We propose that the functions of these glial genes are evolutionarily conserved: their importance to PHP is directly related to their vulnerability to transcriptional dysregulation in AD.

## AUTHOR CONTRIBUTIONS

Tingting Wang conceptualized and designed the project; Tingting Wang and Stefano Vicini supervised the project; Yimei Cai, Tao Cui, and Paxton Paganelli collected data and performed analyses; Pengqi Yin performed data analyses; and Tingting Wang interpreted the data and wrote the manuscript.

## FUNDING INFORMATION

Work in the laboratory of T.T.W. was supported by National Institutes of Health R01 NS117372 (T.T.W.) and R21 NS121284 (T.T.W.), Simons Foundation Autism Research Initiative (SFARI) BTI Award 551354 (T.T.W.), and Brain and Behavior Research Foundation Young Investigator Award 27792 (T.T.W.).

## CONFLICT OF INTEREST STATEMENT

The authors declare no competing interests.

## Supporting information


Figure S1.

Figure S2.

Table S1.

Table S2.

Data S1.

## Data Availability

The published human ChIP‐seq dataset is available at: https://www.synapse.org/#!Synapse:syn4896408. The published human snRNA‐seq dataset is available at https://www.synapse.org/#!Synapse:syn18485175. The published *Drosophila* scRNA‐seq dataset is accessible using GSE107451. All data are available in the main text or the Data S1. All reagents used in the study will be publicly available upon acceptance of the manuscript.

## References

[acel13989-bib-0001] Chipman, P. H. , Fetter, R. D. , Panzera, L. C. , Bergerson, S. J. , Karmelic, D. , Yokoyama, S. , Hoppa, M. B. , & Davis, G. W. (2022). NMDAR‐dependent presynaptic homeostasis in adult hippocampus: Synapse growth and cross‐modal inhibitory plasticity. Neuron, 110, 3302–3317. e3307. 36070750 10.1016/j.neuron.2022.08.014 PMC9588671

[acel13989-bib-0002] Chung, W. S. , Welsh, C. A. , Barres, B. A. , & Stevens, B. (2015). Do glia drive synaptic and cognitive impairment in disease? Nature Neuroscience, 18, 1539–1545. 26505565 10.1038/nn.4142 PMC4739631

[acel13989-bib-0003] Cull‐Candy, S. G. , Miledi, R. , Trautmann, A. , & Uchitel, O. D. (1980). On the release of transmitter at normal, myasthenia gravis and myasthenic syndrome affected human end‐plates. The Journal of Physiology, 299, 621–638. 6103954 10.1113/jphysiol.1980.sp013145 PMC1279245

[acel13989-bib-0004] Davie, K. , Janssens, J. , Koldere, D. , De Waegeneer, M. , Pech, U. , Kreft, L. , Aibar, S. , Makhzami, S. , Christiaens, V. , Bravo Gonzalez‐Blas, C. , Poovathingal, S. , Hulselmans, G. , Spanier, K. I. , Moerman, T. , Vanspauwen, B. , Geurs, S. , Voet, T. , Lammertyn, J. , Thienpont, B. , … Aerts, S. (2018). A single‐cell transcriptome atlas of the aging Drosophila brain. Cell, 174, 982–998. e920. 29909982 10.1016/j.cell.2018.05.057 PMC6086935

[acel13989-bib-0005] Davis, G. W. (2006). Homeostatic control of neural activity: From phenomenology to molecular design. Annual Review of Neuroscience, 29, 307–323. 10.1146/annurev.neuro.28.061604.135751 16776588

[acel13989-bib-0006] Davis, G. W. , & Muller, M. (2015). Homeostatic control of presynaptic neurotransmitter release. Annual Review of Physiology, 77, 251–270. 10.1146/annurev-physiol-021014-071740 25386989

[acel13989-bib-0007] Delvendahl, I. , Kita, K. , & Muller, M. (2019). Rapid and sustained homeostatic control of presynaptic exocytosis at a central synapse. Proceedings of the National Academy of Sciences of the United States of America, 116, 23783–23789. 31685637 10.1073/pnas.1909675116 PMC6876255

[acel13989-bib-0008] Dickman, D. K. , & Davis, G. W. (2009). The schizophrenia susceptibility gene dysbindin controls synaptic homeostasis. Science, 326, 1127–1130. 19965435 10.1126/science.1179685 PMC3063306

[acel13989-bib-0009] Frank, C. A. , Kennedy, M. J. , Goold, C. P. , Marek, K. W. , & Davis, G. W. (2006). Mechanisms underlying the rapid induction and sustained expression of synaptic homeostasis. Neuron, 52, 663–677. 17114050 10.1016/j.neuron.2006.09.029 PMC2673733

[acel13989-bib-0010] Grubman, A. , Chew, G. , Ouyang, J. F. , Sun, G. , Choo, X. Y. , McLean, C. , Simmons, R. K. , Buckberry, S. , Vargas‐Landin, D. B. , Poppe, D. , Pflueger, J. , Lister, R. , Rackham, O. J. L. , Petretto, E. , & Polo, J. M. (2019). A single‐cell atlas of entorhinal cortex from individuals with Alzheimer's disease reveals cell‐type‐specific gene expression regulation. Nature Neuroscience, 22, 2087–2097. 31768052 10.1038/s41593-019-0539-4

[acel13989-bib-0011] Habib, N. , McCabe, C. , Medina, S. , Varshavsky, M. , Kitsberg, D. , Dvir‐Szternfeld, R. , Green, G. , Dionne, D. , Nguyen, L. , Marshall, J. L. , Chen, F. , Zhang, F. , Kaplan, T. , Regev, A. , & Schwartz, M. (2020). Disease‐associated astrocytes in Alzheimer's disease and aging. Nature Neuroscience, 23, 701–706. 32341542 10.1038/s41593-020-0624-8 PMC9262034

[acel13989-bib-0012] Hauswirth, A. G. , Ford, K. J. , Wang, T. , Fetter, R. D. , Tong, A. , & Davis, G. W. (2018). A postsynaptic PI3K‐cII dependent signaling controller for presynaptic homeostatic plasticity. eLife, 7, e31535. 29303480 10.7554/eLife.31535 PMC5773188

[acel13989-bib-0013] Hong, S. , Beja‐Glasser, V. F. , Nfonoyim, B. M. , Frouin, A. , Li, S. , Ramakrishnan, S. , Merry, K. M. , Shi, Q. , Rosenthal, A. , Barres, B. A. , Lemere, C. A. , Selkoe, D. J. , & Stevens, B. (2016). Complement and microglia mediate early synapse loss in Alzheimer mouse models. Science, 352, 712–716. 27033548 10.1126/science.aad8373 PMC5094372

[acel13989-bib-0014] Huffels, C. F. M. , Middeldorp, J. , & Hol, E. M. (2023). Ass pathology and neuron‐glia interactions: A Synaptocentric view. Neurochemical Research, 48, 1026–1046. 35976488 10.1007/s11064-022-03699-6 PMC10030451

[acel13989-bib-0015] Karmodiya, K. , Krebs, A. R. , Oulad‐Abdelghani, M. , Kimura, H. , & Tora, L. (2012). H3K9 and H3K14 acetylation co‐occur at many gene regulatory elements, while H3K14ac marks a subset of inactive inducible promoters in mouse embryonic stem cells. BMC Genomics, 13, 424. 22920947 10.1186/1471-2164-13-424 PMC3473242

[acel13989-bib-0016] Keren‐Shaul, H. , Spinrad, A. , Weiner, A. , Matcovitch‐Natan, O. , Dvir‐Szternfeld, R. , Ulland, T. K. , David, E. , Baruch, K. , Lara‐Astaiso, D. , Toth, B. , Itzkovitz, S. , Colonna, M. , Schwartz, M. , & Amit, I. (2017). A unique microglia type associated with restricting development of Alzheimer's disease. Cell, 169, 1276–1290. e1217. 28602351 10.1016/j.cell.2017.05.018

[acel13989-bib-0017] Klein, H. U. , McCabe, C. , Gjoneska, E. , Sullivan, S. E. , Kaskow, B. J. , Tang, A. , Smith, R. V. , Xu, J. , Pfenning, A. R. , Bernstein, B. E. , Meissner, A. , Schneider, J. A. , Mostafavi, S. , Tsai, L. H. , Young‐Pearse, T. L. , Bennett, D. A. , & de Jager, P. L. (2019). Epigenome‐wide study uncovers large‐scale changes in histone acetylation driven by tau pathology in aging and Alzheimer's human brains. Nature Neuroscience, 22, 37–46. 30559478 10.1038/s41593-018-0291-1 PMC6516529

[acel13989-bib-0018] Knopman, D. S. , Amieva, H. , Petersen, R. C. , Chetelat, G. , Holtzman, D. M. , Hyman, B. T. , Nixon, R. A. , & Jones, D. T. (2021). Alzheimer disease. Nature reviews. Disease primers, 7, 33. 10.1038/s41572-021-00269-y PMC8574196 33986301

[acel13989-bib-0019] Lau, S. F. , Cao, H. , Fu, A. K. Y. , & Ip, N. Y. (2020). Single‐nucleus transcriptome analysis reveals dysregulation of angiogenic endothelial cells and neuroprotective glia in Alzheimer's disease. Proceedings of the National Academy of Sciences of the United States of America, 117, 25800–25809. 32989152 10.1073/pnas.2008762117 PMC7568283

[acel13989-bib-0020] Lord, J. , & Cruchaga, C. (2014). The epigenetic landscape of Alzheimer's disease. Nature Neuroscience, 17, 1138–1140. 25157507 10.1038/nn.3792 PMC5472058

[acel13989-bib-0021] Mathys, H. , Davila‐Velderrain, J. , Peng, Z. , Gao, F. , Mohammadi, S. , Young, J. Z. , Menon, M. , He, L. , Abdurrob, F. , Jiang, X. , Martorell, A. J. , Ransohoff, R. M. , Hafler, B. P. , Bennett, D. A. , Kellis, M. , & Tsai, L. H. (2019). Single‐cell transcriptomic analysis of Alzheimer's disease. Nature, 570, 332–337. 31042697 10.1038/s41586-019-1195-2 PMC6865822

[acel13989-bib-0022] Morabito, S. , Miyoshi, E. , Michael, N. , Shahin, S. , Martini, A. C. , Head, E. , Silva, J. , Leavy, K. , Perez‐Rosendahl, M. , & Swarup, V. (2021). Single‐nucleus chromatin accessibility and transcriptomic characterization of Alzheimer's disease. Nature Genetics, 53, 1143–1155. 34239132 10.1038/s41588-021-00894-z PMC8766217

[acel13989-bib-0023] Muller, M. , Pym, E. C. , Tong, A. , & Davis, G. W. (2011). Rab3‐GAP controls the progression of synaptic homeostasis at a late stage of vesicle release. Neuron, 69, 749–762. 21338884 10.1016/j.neuron.2011.01.025 PMC3059509

[acel13989-bib-0024] Nativio, R. , Donahue, G. , Berson, A. , Lan, Y. , Amlie‐Wolf, A. , Tuzer, F. , Toledo, J. B. , Gosai, S. J. , Gregory, B. D. , Torres, C. , Trojanowski, J. Q. , Wang, L. S. , Johnson, F. B. , Bonini, N. M. , & Berger, S. L. (2018). Dysregulation of the epigenetic landscape of normal aging in Alzheimer's disease. Nature Neuroscience, 21, 497–505. 29507413 10.1038/s41593-018-0101-9 PMC6124498

[acel13989-bib-0025] Orr, B. O. , Hauswirth, A. G. , Celona, B. , Fetter, R. D. , Zunino, G. , Kvon, E. Z. , Zhu, Y. , Pennacchio, L. A. , Black, B. L. , & Davis, G. W. (2020). Presynaptic homeostasis opposes disease progression in mouse models of ALS‐like degeneration: Evidence for homeostatic neuroprotection. Neuron, 107, 95–111. e116. 32380032 10.1016/j.neuron.2020.04.009 PMC7529479

[acel13989-bib-0026] Plomp, J. J. , van Kempen, G. T. , & Molenaar, P. C. (1992). Adaptation of quantal content to decreased postsynaptic sensitivity at single endplates in alpha‐bungarotoxin‐treated rats. The Journal of Physiology, 458, 487–499. 1302275 10.1113/jphysiol.1992.sp019429 PMC1175167

[acel13989-bib-0027] Santana, D. A. , Bedrat, A. , Puga, R. D. , Turecki, G. , Mechawar, N. , Faria, T. C. , Gigek, C. O. , Payao, S. L. , Smith, M. A. , Lemos, B. , & Chen, E. S. (2022). The role of H3K9 acetylation and gene expression in different brain regions of Alzheimer's disease patients. Epigenomics, 14, 651–670. 35588246 10.2217/epi-2022-0096

[acel13989-bib-0028] Selkoe, D. J. (2002). Alzheimer's disease is a synaptic failure. Science, 298, 789–791. 12399581 10.1126/science.1074069

[acel13989-bib-0029] Sierksma, A. , Lu, A. , Mancuso, R. , Fattorelli, N. , Thrupp, N. , Salta, E. , Zoco, J. , Blum, D. , Buee, L. , De Strooper, B. , & Fiers, M. (2020). Novel Alzheimer risk genes determine the microglia response to amyloid‐beta but not to TAU pathology. EMBO Molecular Medicine, 12, e10606. 31951107 10.15252/emmm.201910606 PMC7059012

[acel13989-bib-0030] Skene, N. G. , & Grant, S. G. (2016). Identification of vulnerable cell types in major brain disorders using single cell transcriptomes and expression weighted cell type enrichment. Frontiers in Neuroscience, 10, 16. 26858593 10.3389/fnins.2016.00016 PMC4730103

[acel13989-bib-0031] Stogsdill, J. A. , & Eroglu, C. (2017). The interplay between neurons and glia in synapse development and plasticity. Current Opinion in Neurobiology, 42, 1–8. 27788368 10.1016/j.conb.2016.09.016 PMC5316301

[acel13989-bib-0032] Styr, B. , & Slutsky, I. (2018). Imbalance between firing homeostasis and synaptic plasticity drives early‐phase Alzheimer's disease. Nature Neuroscience, 21, 463–473. 29403035 10.1038/s41593-018-0080-x PMC6533171

[acel13989-bib-0033] Tasaki, S. , Gaiteri, C. , Petyuk, V. A. , Blizinsky, K. D. , De Jager, P. L. , Buchman, A. S. , & Bennett, D. A. (2019). Genetic risk for Alzheimer's dementia predicts motor deficits through multi‐omic systems in older adults. Translational Psychiatry, 9, 241. 31582723 10.1038/s41398-019-0577-4 PMC6776503

[acel13989-bib-0034] Turrigiano, G. G. (2008). The self‐tuning neuron: Synaptic scaling of excitatory synapses. Cell, 135, 422–435. 18984155 10.1016/j.cell.2008.10.008 PMC2834419

[acel13989-bib-0035] Wang, T. , Hauswirth, A. G. , Tong, A. , Dickman, D. K. , & Davis, G. W. (2014). Endostatin is a trans‐synaptic signal for homeostatic synaptic plasticity. Neuron, 83, 616–629. 25066085 10.1016/j.neuron.2014.07.003 PMC4133507

[acel13989-bib-0036] Wang, T. , Morency, D. T. , Harris, N. , & Davis, G. W. (2020). Epigenetic signaling in glia controls presynaptic homeostatic plasticity. Neuron, 105, 491–505. e493. 31810838 10.1016/j.neuron.2019.10.041 PMC7518042

[acel13989-bib-0037] Wang, X. , McIntosh, J. M. , & Rich, M. M. (2018). Muscle nicotinic acetylcholine receptors may mediate trans‐synaptic signaling at the mouse neuromuscular junction. The Journal of Neuroscience, 38, 1725–1736. 29326174 10.1523/JNEUROSCI.1789-17.2018 PMC5815454

[acel13989-bib-0038] Wondolowski, J. , & Dickman, D. (2013). Emerging links between homeostatic synaptic plasticity and neurological disease. Frontiers in Cellular Neuroscience, 7, 223. 24312013 10.3389/fncel.2013.00223 PMC3836049

[acel13989-bib-0039] Yildirim, K. , Petri, J. , Kottmeier, R. , & Klambt, C. (2019). Drosophila glia: Few cell types and many conserved functions. Glia, 67, 5–26. 30443934 10.1002/glia.23459

[acel13989-bib-0040] Yin, P. , Cai, Y. , Cui, T. , Berg, A. J. , Wang, T. , Morency, D. T. , Paganelli, P. M. , Lok, C. , Xue, Y. , Vicini, S. , & Wang, T. (2023). Glial sphingosine‐mediated epigenetic regulation stabilizes synaptic function in drosophila models of Alzheimer's disease. Journal of Neuroscience. 10.1523/JNEUROSCI.0515-23.2023 [In press]. PMC10586542 37669862

[acel13989-bib-0041] Zeng, H. , Huang, J. , Zhou, H. , Meilandt, W. J. , Dejanovic, B. , Zhou, Y. , Bohlen, C. J. , Lee, S. H. , Ren, J. , Liu, A. , Tang, Z. , Sheng, H. , Liu, J. , Sheng, M. , & Wang, X. (2023). Integrative in situ mapping of single‐cell transcriptional states and tissue histopathology in a mouse model of Alzheimer's disease. Nature Neuroscience, 26, 430–446. 36732642 10.1038/s41593-022-01251-x PMC11332722

[acel13989-bib-0042] Zhou, Y. , Song, W. M. , Andhey, P. S. , Swain, A. , Levy, T. , Miller, K. R. , Poliani, P. L. , Cominelli, M. , Grover, S. , Gilfillan, S. , Cella, M. , Ulland, T. K. , Zaitsev, K. , Miyashita, A. , Ikeuchi, T. , Sainouchi, M. , Kakita, A. , Bennett, D. A. , Schneider, J. A. , … Colonna, M. (2020). Human and mouse single‐nucleus transcriptomics reveal TREM2‐dependent and TREM2‐independent cellular responses in Alzheimer's disease. Nature Medicine, 26, 131–142. 10.1038/s41591-019-0695-9 PMC6980793 31932797

