# Supplementary figures and images for: Dysregulated glial genes in Alzheimer's disease are essential for homeostatic plasticity: Evidence from integrative epigenetic and single cell analyses
Source: Aging Cell. 2023 Sep 15;22(11):e13989. doi: 10.1111/acel.13989 (PMC10652298; doi:10.1111/acel.13989)

(a)

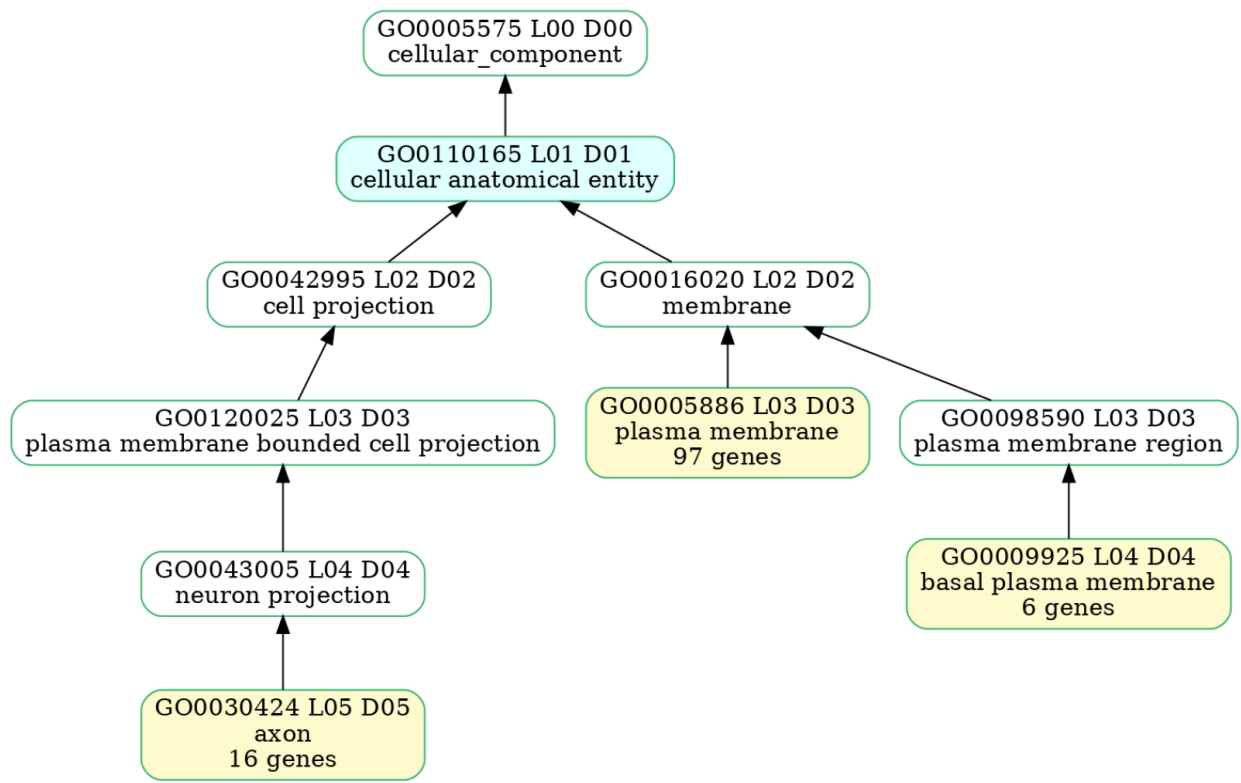

(b)

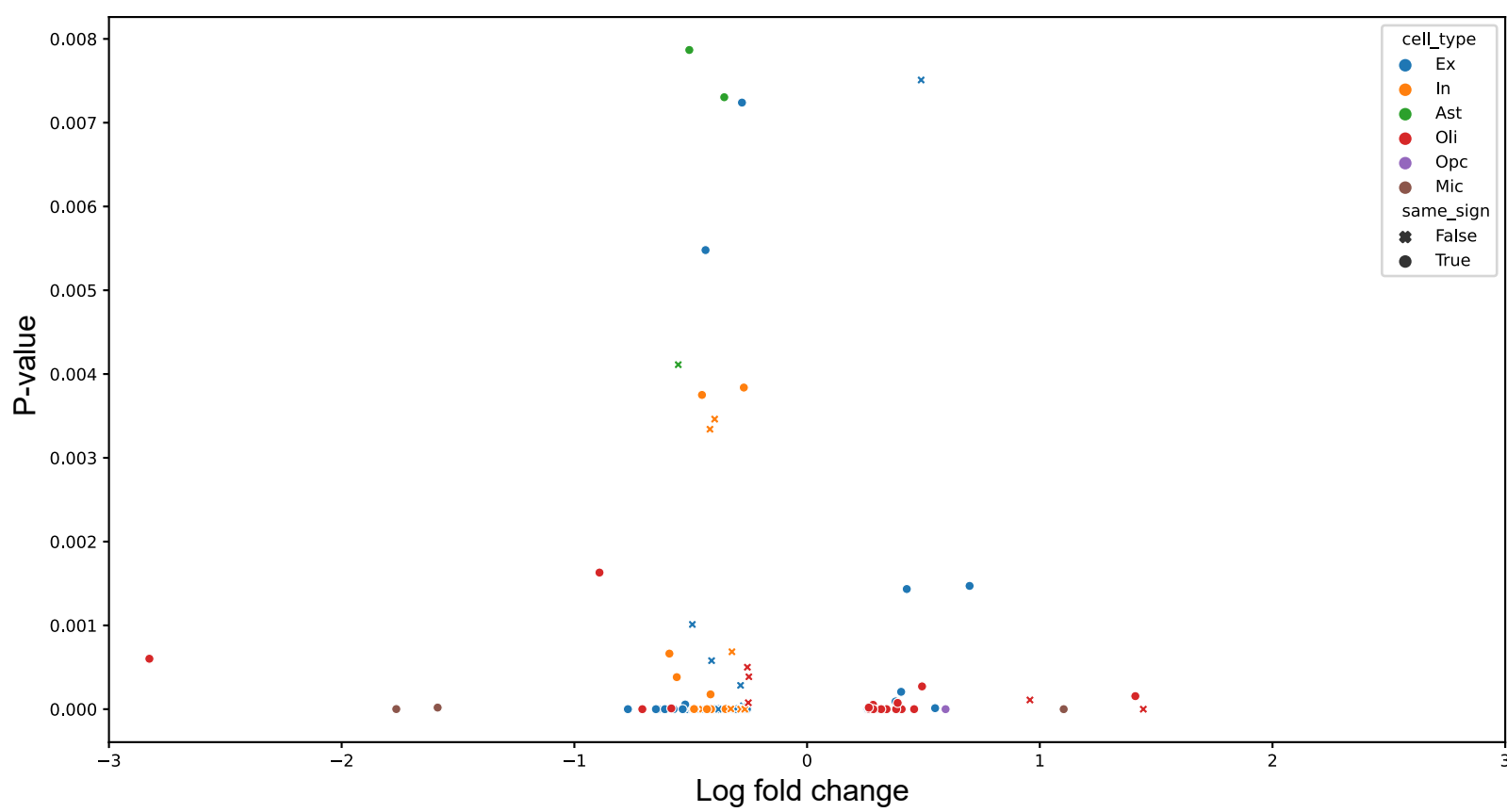

Supplement: Supplementary file 1 — Figure S1. [file ACEL-22-e13989-s005.pdf]

(a)

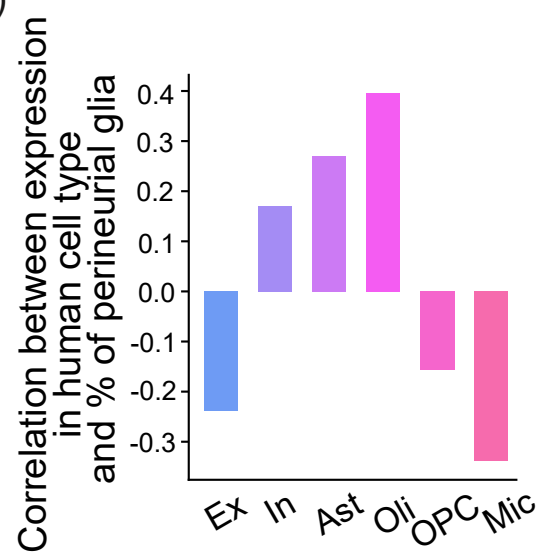

(b)

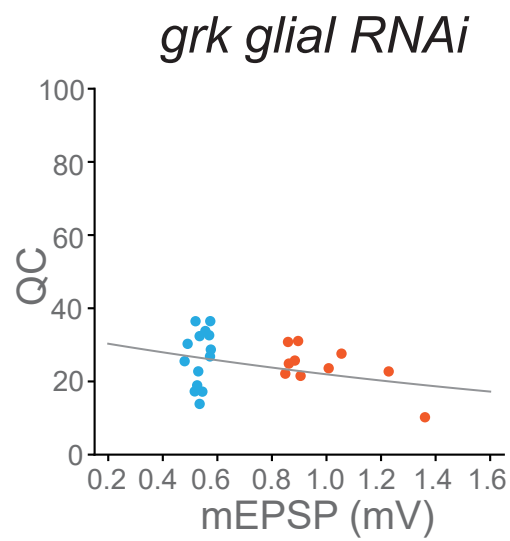

Supplement: Supplementary file 2 — Figure S2. [file ACEL-22-e13989-s004.pdf]
