# Supplementary material for: Dysregulated glial genes in Alzheimer's disease are essential for homeostatic plasticity: Evidence from integrative epigenetic and single cell analyses
Source: Aging Cell. 2023 Sep 15;22(11):e13989. doi: 10.1111/acel.13989 (PMC10652298; doi:10.1111/acel.13989)
Supplement: Supplementary file 5 — Data S1. [file ACEL-22-e13989-s001.docx]

**SUPPLEMENTARY MATERIAL**

Dysregulated Glial Genes in Alzheimer’s Disease Are Essential for Homeostatic Plasticity: Evidence from Integrative Epigenetic and Single Cell Analyses

Yimei Cai^1,†^, Tao Cui^1,2,†^, Pengqi Yin^1,3,4^, Paxton Paganelli^1^, Stefano Vicini^1,2^, Tingting Wang^1,2,*^

^1^Department of Pharmacology & Physiology, Georgetown University Medical Center, Washington, D.C. 20007, USA.

^2^Interdisciplinary Program in Neuroscience, Georgetown University Medical Center, Washington, D.C. 20007, USA.

^3^Current address: Department of Neurology, Shanghai General Hospital, Shanghai Jiao Tong University School of Medicine, Shanghai 200080, China

^4^Department of Neurology, First Affiliated Hospital, Harbin Medical University, Harbin 150081, China

*Correspondence: [tw652@georgetown.edu](mailto:tw652@georgetown.edu)

† These authors contributed equally to this work

**Supplementary Methods**

***Drosophila melanogaster* strains and husbandry**

*Drosophila* stocks were raised at room temperature on standard molasses food. *Drosophila* alleles used for experiments were raised at 25°C. Unless otherwise noted, the *w1118* strain was used as a *wild-type* (*wt*) control. All genetic mutants and RNAi alleles (see list below for details) are from Bloomington *Drosophila* Stock Center (BDRC). *Drosophila* homologues of human genes were determined using flybase.org as of 02/15/2022.

***Drosophila* lines used in the study:**

| *Drosophila* lines | Source | Identifier |
| --- | --- | --- |
| *Septin5* | Bloomington | BL91000 (II) |
| *CG42709* | Bloomington | BL16816 (III) |
| *h* | Bloomington | BL513 (III) |
| *UAS-h RNAi* | Bloomington | BL34326 (III) |
| *dpn* | Bloomington | BL30603 (II) |
| *UAS-dpn RNAi* | Bloomington | BL26320 (III) |
| *E2f1* | Bloomington | BL20929 (III) |
| *UAS-E2f1* | Bloomington | BL34058 (III) |
| *NimC1* | Bloomington | BL59190 (II) |
| *UAS-NimC1 RNAi* | Bloomington | BL25787 (III) |
| *M6* | Bloomington | BL12416 (III) |
| *UAS-M6 RNAi* | Bloomington | BL54032 (II) |
| *UAS-grk RNAi* | Bloomington | BL55926 (II) |
| *Mkp3* | Bloomington | BL36023 (III) |
| *Cep97* | Bloomington | BL27930 (II) |
| *NP6293-Gal4* | (Stork et al., 2012; Wang et al., 2020) | Marc Freeman (Vollum Institute, Portland, Oregon) (II) |
| *Repo-Gal4* | (Stork et al., 2012; Wang et al., 2020) | Marc Freeman (Vollum Institute, Portland, Oregon) (II) |
| *Ok371-Gal4* | Bloomington | BL26160 (II) |

**ChIP-seq, snRNA-seq, and GO analysis**

GO enrichment analysis was done using Python library GOATOOLS version 1.2.3 (Klopfenstein et al., 2018). For cross comparisons of the H3K9ac ChIP-seq (Klein et al., 2019) and snRNA-seq (Mathys et al., 2019) datasets, we used H3K9ac target genes (associated with Aβ) that were annotated by chromatin states as in the published study. In total, 87 unique genes (100 genes if repetitive DEGs in different cell types are counted) are both H3K9ac targets and DEGs. Among these genes, 69 DEGs (59 unique genes) have the “same sign” between Coefficient Aβ and transcriptional log_2_fold change; 23 DEGs are up-regulated and 46 are DEGs. *Drosophila* brain scRNA-seq (Davie et al., 2018) and human brain snRNA-seq (non-pathological, (Mathys et al., 2019)) datasets are used for gene enrichment and correlation analysis. Pearson (scipy.stats.pearsonr) and spearman (scipy.stats.spearmanr) correlation analysis and statistical tests are shown in the results or Figure legends. All ChIP-seq, snRNA-seq, scRNA-seq, gene enrichment, and correlation analysis was performed using custom scripts in Python 3.7.12.

**Electrophysiology**

Sharp-electrode recordings were made from muscle 6 at abdominal segments 2 and 3 in third-instar larvae using an Axoclamp 900A amplifier (Molecular Devices). HL3 saline was used (in mM): 70 NaCl, 5 KCl, 10 MgCl2, 10 NaHCO3, 115 Sucrose, 5 Trehalose, 5 HEPES, and 0.3 CaCl2. EPSP and mEPSP traces were analyzed in Stimfit (Python) with previously published routines and MiniAnalysis (Synaptosoft). For the rapid induction of synaptic homeostasis, larvae were incubated in 20µM Philanthotoxin-433 in an un-stretched, partially dissected preparation (PhTX, Aobious) for 10min (Dickman and Davis, 2009; Frank et al., 2006). For each NMJ, the average amplitudes of evoked EPSP are based on the mean peak amplitudes in response to 20-30 individual stimuli. Spontaneous mEPSPs were recorded continuously 60-90s. Quantal content was estimated for each NMJ as the ratio of EPSP amplitude/mEPSP amplitude. The mean value across all NMJ for a given genotype is reported.

**Statistical analysis**

Quantification of data are presented as Mean ± Standard Error of the Mean (SEM) with the precise sample size indicated in the Figure legends or the Supplementary Figure legends. Statistical analysis was performed using Python (3.7.12) and Prism (9.4.1, GraphPad). We used unpaired two-tailed Student’s t test as indicated in the Results or Figure legends. We also ran the Mann-Whitney U test on all the data and the significance remains the same.

**Supplementary Figure Legends**

**Figure S1. GO Analysis for H3K9ac-target Genes Associated with Aβ.**

(**a**) GO terms identified from genes, annotated by chromatin states as in the published study, which are associated with Aβ, referring directly to data presented in Figure 1b.

(**b**) P-values for transcriptional log fold change for genes that are both H3K9ac-target genes associated with Aβ and also cell type-specific DEGs in excitatory neuron (Ex), inhibitory neuron (In), astrocyte (Ast), oligodendrocyte (Oli), oligodendrocyte progenitor cell (OPC), and microglia (Mic). Genes that have Coefficient Aβ and log fold change with the “same sign” are shown with dots.

**Figure S2. Analysis of Perineurial Glia Expressing “Synaptic” Genes and PHP Impairment in *Grk* Glial Knockdown.**

**(a)** The Spearman correlation between the expression of human “synaptic” genes in various cell types and the percentage of perineurial glia that express the human genes’ homologues in *Drosophila*.

(**b**) The relationship between quantal content (QC) and mEPSP amplitude in the presence (+PhTX) and the absence of philanthotoxin (-PhTX) is shown for *NP6293-Gal4>UAS-grk-RNAi* (*grk glial RNAi*). Data points in the plot represent individual cells. Exponential fit line for the datapoints is shown in gray.

**Supplementary Tables**

**Table S1. DEGs Have the Same Sign in Coefficient Aβ and Log Fold Change.**

**Table S2. DEGs Have Different Signs in Coefficient Aβ and Log Fold Change.**

**References**

Davie, K., Janssens, J., Koldere, D., De Waegeneer, M., Pech, U., Kreft, L., Aibar, S., Makhzami, S., Christiaens, V., Bravo Gonzalez-Blas, C.*, et al.* (2018). A Single-Cell Transcriptome Atlas of the Aging Drosophila Brain. Cell *174*, 982-998 e920.

Dickman, D.K., and Davis, G.W. (2009). The schizophrenia susceptibility gene dysbindin controls synaptic homeostasis. Science *326*, 1127-1130.

Frank, C.A., Kennedy, M.J., Goold, C.P., Marek, K.W., and Davis, G.W. (2006). Mechanisms underlying the rapid induction and sustained expression of synaptic homeostasis. Neuron *52*, 663-677.

Klein, H.U., McCabe, C., Gjoneska, E., Sullivan, S.E., Kaskow, B.J., Tang, A., Smith, R.V., Xu, J., Pfenning, A.R., Bernstein, B.E.*, et al.* (2019). Epigenome-wide study uncovers large-scale changes in histone acetylation driven by tau pathology in aging and Alzheimer's human brains. Nat Neurosci *22*, 37-46.

Klopfenstein, D.V., Zhang, L., Pedersen, B.S., Ramirez, F., Warwick Vesztrocy, A., Naldi, A., Mungall, C.J., Yunes, J.M., Botvinnik, O., Weigel, M.*, et al.* (2018). GOATOOLS: A Python library for Gene Ontology analyses. Sci Rep *8*, 10872.

Mathys, H., Davila-Velderrain, J., Peng, Z., Gao, F., Mohammadi, S., Young, J.Z., Menon, M., He, L., Abdurrob, F., Jiang, X.*, et al.* (2019). Single-cell transcriptomic analysis of Alzheimer's disease. Nature *570*, 332-337.

Stork, T., Bernardos, R., and Freeman, M.R. (2012). Analysis of glial cell development and function in Drosophila. Cold Spring Harb Protoc *2012*, 1-17.

Wang, T., Morency, D.T., Harris, N., and Davis, G.W. (2020). Epigenetic Signaling in Glia Controls Presynaptic Homeostatic Plasticity. Neuron *105*, 491-505 e493.
